# Incorporating Genome Annotation Into Genomic Prediction for Carcass Traits in Chinese Simmental Beef Cattle

**DOI:** 10.3389/fgene.2020.00481

**Published:** 2020-05-15

**Authors:** Ling Xu, Ning Gao, Zezhao Wang, Lei Xu, Ying Liu, Yan Chen, Lingyang Xu, Xue Gao, Lupei Zhang, Huijiang Gao, Bo Zhu, Junya Li

**Affiliations:** ^1^Laboratory of Molecular Biology and Bovine Breeding, Institute of Animal Sciences, Chinese Academy of Agricultural Sciences, Beijing, China; ^2^State Key Laboratory of Biocontrol, School of Life Sciences, Sun Yat-sen University, Guangzhou, China; ^3^National Centre of Beef Cattle Genetic Evaluation, Beijing, China

**Keywords:** genomic prediction, genome annotation, haplotype, Chinese Simmental beef cattle, prediction accuracy

## Abstract

Various methods have been proposed for genomic prediction (GP) in livestock. These methods have mainly focused on statistical considerations and did not include genome annotation information. In this study, to improve the predictive performance of carcass traits in Chinese Simmental beef cattle, we incorporated the genome annotation information into GP. Single nucleotide polymorphisms (SNPs) were annotated to five genomic classes: intergenic, gene, exon, protein coding sequences, and 3′/5′ untranslated region. Haploblocks were constructed for all markers and these five genomic classes by defining a biologically functional unit, and haplotype effects were modeled in both numerical dosage and categorical coding strategies. The first-order epistatic effects among SNPs and haplotypes were modeled using a categorical epistasis model. For all makers, the extension from the SNP-based model to a haplotype-based model improved the accuracy by 5.4–9.8% for carcass weight (CW), live weight (LW), and striploin (SI). For the five genomic classes using the haplotype-based prediction model, the incorporation of gene class information into the model improved the accuracies by an average of 1.4, 2.1, and 1.3% for CW, LW, and SI, respectively, compared with their corresponding results for all markers. Including the first-order epistatic effects into the prediction models improved the accuracies in some traits and genomic classes. Therefore, for traits with moderate-to-high heritability, incorporating genome annotation information of gene class into haplotype-based prediction models could be considered as a promising tool for GP in Chinese Simmental beef cattle, and modeling epistasis in prediction can further increase the accuracy to some degree.

## Introduction

Genomic prediction (GP), which uses whole-genome markers to predict genomic breeding value, has been widely used in breeding programs of plants (Heffner et al., 2009; Riedelsheimer et al., 2012; de los Campos et al., 2013; Hayes et al., 2013) and domestic animals (Sonesson and Meuwissen, 2009; Hayes et al., 2010; Erbe et al., 2012; de los Campos et al., 2013), disease risk prediction for humans (Vazquez et al., 2012; Akey et al., 2014; Abraham et al., 2016), and phenotype prediction of model organisms (Ober et al., 2012; Kooke et al., 2016). Accompanied by the fast development of genotyping and sequencing technologies, various methods with different underlying statistical assumptions have been proposed for GP, including penalized and Bayesian regression methods (Whittaker et al., 2000; Meuwissen et al., 2001; Gianola et al., 2006; VanRaden, 2008; Bennewitz et al., 2009; Habier et al., 2011; Gianola, 2013; Morota and Gianola, 2014). These methods have been applied in cattle populations to improve the prediction accuracy of direct genomic estimated breeding values (DGVs) to some degree (Luan et al., 2009; Hayes et al., 2010; Bolormaa et al., 2013; Fernandes Júnior et al., 2016; Mehrban et al., 2017; Toghiani et al., 2017). However, these established prediction methods have mainly focused on statistical considerations and did not consider the abundantly available biological information. Incorporating biological knowledge, like annotation information (Gao et al., 2017) and gene expression (Li et al., 2019), into GP using an appropriate method may bridge the gap between mathematical models and the underlying biological processes; thus, this information has the potential to improve the prediction accuracy under certain circumstances (Edwards et al., 2016).

Given the availability of genome annotation information, some studies have tried to integrate this information into prediction models to improve the predictive accuracies (Morota et al., 2014; Do et al., 2015; Abdollahi-Arpanahi et al., 2016; Gao et al., 2017; Nani et al., 2019). Single nucleotide polymorphisms (SNPs) were divided into different genomic classes based on the genome annotation information, and GP was conducted for genomic classes using two strategies. The first strategy was to assess the prediction accuracy for each genomic class, and then the genomic class that give the best prediction accuracy was further used for GP (Morota et al., 2014; Do et al., 2015; Abdollahi-Arpanahi et al., 2016). Another strategy was to assign different prior distributions for the different genomic classes, and then all genomic classes were used for prediction (MacLeod et al., 2016). These approaches for incorporating annotation information into GP slightly improved the prediction accuracy in some cases. For instance, Erbe et al. (2012) found that SNPs in the transcribed class produce better predictive performance than other classes in dairy cattle, with a slight increase in prediction accuracy of 0.03 for milk yield, fat yield, and protein yield traits on average. However, others discovered that the prediction accuracy of genomic classes was trait-dependent in the commercial chicken population, and the predictive performance of the whole-genome region remained more accurate (Morota et al., 2014). Generally, these studies have not achieved significant improvements over their corresponding predictions without annotation information. Most studies simply applied standard prediction models for genomic classes based on individual SNPs, with the basic underlying assumption is that at least one marker is in linkage disequilibrium (LD) with each quantitative trait locus (QTL) under high-density markers. The marker density of genomic classes declined after the partitioning, which caused fewer bi-allelic SNPs in LD with a QTL.

An alternative is treating haplotypes that are on tuples of SNPs as predictor variables in GP to compensate for the imperfect LD between SNPs and QTLs (Cuyabano et al., 2015; Da, 2015). The main benefit of using haplotypes for GP is that a haplotype is expected to have a higher LD with a QTL than an individual marker (Calus et al., 2008), and has better ability to identify mutations than a single SNP (Cuyabano et al., 2014). For a trait controlled by rare QTLs, the fitting haplotype could yield a higher accuracy, regardless of the minor allele frequency (MAF) of the QTL (de los Campos et al., 2013). When a high-density SNPs chip was annotated into different genomic classes, at least two SNPs may be included in a genome feature; thus, multi-allelic haplotype-based prediction models are expected to capture the state of a QTL better than single-SNP-based prediction models for genomic classes (Calus et al., 2008; Meuwissen et al., 2014).

In this study, we used annotation information of the cattle genome to divide Illumina BovineHD BeadChip into five genomic classes, including intergenic regions (IGR), gene, exon, protein coding sequences (CDS), and 3′/5′ untranslated regions (UTR) classes. Then, haploblocks were created (Meuwissen et al., 2014) and haplotype effects were modeled using both numerical dosage and categorical coding strategies (Martini et al., 2017) for each genomic class. Although an additive model may explain a major part of the genetic variance in different datasets (Hill et al., 2008), this model does not explicitly capture any kind of interaction that may be present in biochemical pathways that connect gene expression with the ultimate target phenotype. Therefore, statistical models that incorporate interactions between loci are considered as potentially beneficial for GP (Palucci et al., 2007; Pettersson et al., 2011; Su et al., 2012; Mackay, 2014). Epistasis resulting from interactions between genes at different loci was recognized as an important component in dissecting genetic pathways and understanding the evolution of complex genetic systems (Phillips, 2008; Jiang and Reif, 2015). Overall, the objectives of this study were (1) to compare the predictive accuracies of haplotype-based prediction models with SNP-based prediction models, (2) to characterize the predictive performance when genome annotation information was incorporated into haplotype-based prediction model, and (3) to investigate the contribution of epistasis for the accuracy of GP for carcass traits in Chinese Simmental beef cattle.

## Materials and Methods

### Data

Our dataset includes 1346 Simmental cattle born between 2008 and 2015 from Ulgai, Xilingol League, and Inner Mongolia, China. After weaning, cattle were moved to Jinweifuren Co., Ltd. (Beijing, China) for fattening under the same feeding and management conditions. A more detailed description of the management processes was reported in previous studies (Zhu et al., 2016, 2017). All individuals were slaughtered at an average age of 20 months, and carcass and meat quality traits were measured in accordance with the guidelines proposed by the Institutional of Meat Purchase Specifications. All animals used in the study were treated following the guidelines established by the Council of China Animal Welfare. Protocols of the experiments were approved by the Science Research Department of the Institute of Animal Sciences, Chinese Academy of Agricultural Sciences (CAAS) (Beijing, China). The approval ID/permit numbers are SYXK (Beijing) 2008-007 and SYXK (Beijing) 2008-008. In our study, carcass weight (CW), live weight (LW), and striploin (SI) were analyzed, and their statistical description was summarized in Table 1.

**TABLE 1:** Statistical description and heritability estimation of three traits in Chinese Simmental beef cattle.

Traits^1^	The number of phenotype	Mean (SD)	Maximum	Minimum	*h*^2^(SE)
CW	1346	270.67 ± 45.20	486.00	162.60	0.42 ± 0.05
LW	1342	504.95 ± 70.22	776.00	318.00	0.38 ± 0.07
SI	1342	8.55 ± 1.99	15.90	3.21	0.40 ± 0.05

*^1^Carcass weight (CW), live weight (LW), and striploin (SI).*

### Genotyping and Quality Control

The DNA for each animal was obtained from blood using routine procedures. Samples were genotyped with Illumina BovineHD BeadChip. This array contains 777,962 SNPs with an average probe spacing of 3.43 kb and a median spacing of 2.68 kb. Before statistical analysis, the original SNP dataset was filtered using PLINK (v1.90) (Purcell et al., 2007; Chang et al., 2015). Individuals and autosomal SNPs that failed in any of the following criteria were removed, SNPs call rate (>0.90) (MAF > 0.01), Hardy–Weinberg Equilibrium (*p* > 10^–6^) and individual call rate (>0.90). Missing genotypes were imputed using BEAGLE (v4.1) (Browning and Browning, 2016). Consequently, 1331 individuals and 671,204 SNPs remained. SNPs were coded as the number of copies of the minor allele, i.e., 0, 1, and 2 for the first homozygote, the heterozygote, and the second homozygote, respectively. About population structure, like principal component analysis (PCA) and linkage disequilibrium (LD) were performed in previous studies, which have shown that this population could be separated into five clusters, and the LD (r^2^) dropped below 0.2 at distances of 34 kb, indicating that the implementation of GS in this population requires at least 77,941 markers (Niu et al., 2016; Xia et al., 2016).

### Heritability Estimation

Phenotypes were adjusted for the environmental fixed effects, including sex, year, and the covariates of body weight upon entering the fattening farm, and the number of fattening days. Subsequently, the adjusted phenotypes were used for further analysis. Variance components were estimated using the following univariate animal model in ASREML (v4.1) (Gilmour et al., 2015):

(1) $$y=1_{n}\,\mu +Z\,a+e$$

where *y* is the vector of the adjusted phenotypes, 1_n_is an *n* × 1 vector with entries equal to 1; μ is the overall mean; $a∼N\,\left(0,\sigma_{\text{a}}^{2}\,G\right)$ is a vector of random additive genetic effect, where *G* is the additive genetic relationship matrix constructed using all SNPs and $\sigma_{\text{a}}^{2}$ is the additive genetic variance, *Z* is incidence matrix associating *a*; and $e∼N\,\left(0,\sigma_{\text{e}}^{2}\,I\right)$ is a vector of random residuals, where *I* is the identity matrix and $\sigma_{\text{e}}^{2}$ is the residual variance. The heritability of each trait was estimated using h2=σa2/(σa2+σ)e2.

### SNP Annotation

The latest bovine genome annotation (Bos_taurus.ARS-UCD1.2) was downloaded from Ensemble^1^. According to genome annotation information, the bovine genome was partitioned into five genomic classes: (1) intergenic regions (IGR), (2) gene, (3) exon, (4) protein coding sequences (CDS), and (5) 3′/5′ untranslated regions (UTR) classes. Gene class contained the exon class, and exon class represented a combination of CDS and UTR classes. Thus, overlapping existed among different genomic classes. Then, the SNPs of BovineHD Beadchip were annotated into the corresponding genomic class based on their physical position.

### Haplotype Derivation and Encoding

For the gene, exon, CDS, and UTR classes, a genome feature refers to a single gene, exon, CDS, and UTR, respectively; for the IGR class, a genome feature refers to an interval between two adjacent genes. A group of SNPs that were annotated in a certain genome feature of the five genomic classes was called an SNP set. The phased consecutive SNPs were used for haploblock construction via the approach described by Meuwissen et al. (2014) for each SNP set. The number of SNPs contained in each haploblock depends on the predefined number of types for haplotype allele configurations; here, we used 10 as the maximum number of types (Meuwissen et al., 2014). For SNP sets containing only one SNP, the 0-, 1-, or 2-encoded genotypes were retained for further analysis. Subsequently, haploblocks with at least two haplotype alleles were generated for each SNP set of different genomic classes.

Haplotype effects were then modeled using both numerical dosage (Calus et al., 2008; Cuyabano et al., 2014; Meuwissen et al., 2014) and categorical (Martini et al., 2017) coding strategies. In the numerical dosage model, pseudo-markers were generated for haploblocks by counting the number of copies of the respective allele carried by a certain individual, where the intra-locus additive effects were assumed. The additivity assumption was not necessary in the categorical coding, where the pseudo-markers of haploblocks were coded according to the haplotype allele configurations (genotypes), and each haplotype allele had its own independent effect. Table 2 shows the coding of a haplotype formed by two consecutive SNPs. Thus, for the five genomic classes, the pseudo-marker matrixes with entries 0, 1, and 2 were reconstructed in both numerical dosage and categorical models (CMs). For all markers, haploblocks were constructed for each chromosome separately using the same approach described above, and the process started from the first marker and followed by their physical order, whereas the genome annotation information was not used to define a biologically functional unit.

**TABLE 2:** Numerical and categorical coding of a haploblock formed by two consecutive single nucleotide polymorphisms (SNPs).

Haplotype allele 1	Haplotype allele 2	Categorical coding of haploblock^1^	Numerical coding of haploblock			
			AB	Ab	aB	ab
AB	AB	AB|AB	2	0	0	0
AB	Ab	AB|Ab	1	1	0	0
AB	aB	AB|aB	1	0	1	0
AB	ab	AB|ab	1	0	0	1
Ab	Ab	Ab|Ab	0	2	0	0
Ab	aB	Ab|aB	0	1	1	0
Ab	ab	Ab|ab	0	1	0	1
aB	aB	aB|aB	0	0	2	0
aB	ab	aB|ab	0	0	1	1
ab	ab	ab|ab	0	0	0	2

*^1^separates the strands of DNA. Considering this haploblock (let {A, a} and {B, b} denote alleles harbored by the two SNPs, respectively), four possible types of gametes—AB, Ab, aB, and ab—could be generated and 10 types of genotypes are possibly formed in a large population (imprinting is not considered).*

### Prediction Models

The prediction model used in this study was basically the same as in Eq. (1), except for the different genomic relatedness matrices **G**, which were constructed based on respective prediction approaches (Table 3). In our study, the predictive accuracies of using all markers were considered as a benchmark.

**TABLE 3:** Genomic relatedness matrices for different genomic prediction models for all markers or haplotypes.

Models	Description	Relatedness matrices	Use^1^
*GBLUP*	Genomic best linear unbiased prediction	$G=\frac{\left(M-P\right)\,{\left(M-P\right)}^{\prime }}{2\,\sum_{i-1}^{m}p_{i}\,\left(1-p_{i}\right)}$	All markers
*G*_*H*_*BLUP*	Haplotype based GBLUP	$G_{H}=\frac{M_{H}\,M_{H}^{\prime }}{Q_{H}}$	All markers
*G*_*H*_*BLUP*|*GA*	Haplotype based *GBLUP* given genome annotation	$G_{H_{\text{GA}}}=\frac{M_{H_{\text{GA}}}\,M_{H_{\text{GA}}}^{\prime }}{Q_{H_{\text{GA}}}}$	Genomic classes
CM	Categorical marker effect model	$S=\left(\frac{\sum_{q-1}^{Q}\varphi_{j\,i\,k}}{m}\right)\,i\,j$	All markers
CE	Categorical epistasis model	*E* = 0.5 × *mS*#(*mS* + 1_*n*×*n*_)/*m*^2^	All markers
*C*_*H*_*M*	Haplotype based CM	$S_{H}=\left(\frac{\sum_{q-1}^{Q}\varphi_{j\,i\,q}}{Q_{H}}\right)\,i\,j$	All markers
*C*_*H*_*E*	Haplotype based CE	$E_{H}=\frac{0.5\times Q_{H}\,S_{H}\,\#\,\left(Q_{H}\,S_{H}+1_{n\times n}\right)}{Q_{H}^{2}}$	All markers
*C*_*H*_*M*|*GA*	*C*_*H*_*M* given genome annotation	$\tilde{S}=\left(\frac{\sum_{q-1}^{Q}\varphi_{j\,i\,q}}{Q_{H_{\text{GA}}}}\right)\,i\,j$	Genomic classes
*C*_*H*_*E*|*GA*	*C*_*H*_*E* given genome annotation	$\tilde{E}=0.5\times Q\,\tilde{S}\,\#\,\left(Q\,\tilde{S}+1_{n\times n}\right)/Q_{H_{\text{GA}}}^{2}$	Genomic classes

*^1^Refers to the whole genome-wide SNP; genomic classes refer to IGR, gene, exon, CDS, and UTR class.*

In numerical dosage models, GBLUP (VanRaden, 2008) was performed for all markers, and the genomic relatedness matrix was calculated as $G=\frac{\left(M-P\right)\,{\left(M-P\right)}^{\prime }}{2\,\sum_{i-1}^{m}p_{\text{i}}\,\left(1-p_{\text{i}}\right)}$, where **M** denotes the (0, 1, and 2) encoded genotype matrix, p_i_ is the MAF of marker i, m is the number of markers, and P is a matrix with columns equal to 2p_i_. The haplotype-based genomic best linear unbiased prediction (*G*_H_*BLUP*) was performed for all markers. The haplotype-based genomic relatedness matrix in *G*_H_*BLUP* was constructed as the dot product of the haplotype allele matrix (M_H_) and expressed as $G_{\text{H}}=\frac{M_{\text{H}}\,M_{\text{H}}^{\prime }}{Q_{\text{H}}}$, where M_H_ is the pseudo-markers matrix with entries 0, 1, and 2 representing the number of copies of each haplotype allele in a haploblock, and Q_H_ is the total number of haploblocks of whole genome.

For the five genomic classes, haplotype-based genomic best linear unbiased prediction given genome annotation (*G*_H_*BLUP*|*GA*) was implemented. Similarly, the haplotype-based genomic relatedness matrix in *G*_H_*BLUP*|*GA* was constructed as $G_{H_{\text{GA}}}=\frac{M_{H_{\text{GA}}}\,M_{H_{\text{GA}}}^{\prime }}{Q_{H_{\text{GA}}}}$, where $M_{H_{\text{GA}}}$ is the haplotype allele matrix with pseudo-markers encoded with (0, 1, and 2), and $Q_{H_{\text{GA}}}$ is the total number of haploblocks in the corresponding genomic class.

In CMs, the SNP-based CM (Martini et al., 2017) was applied for all markers, and the genomic relatedness matrix in CM is expressed as S with entries $S_{\text{ij}}=\frac{\sum_{q-1}^{\text{Q}}\varphi_{\text{jik}}}{m}$, in which φ_jik_ was scored 1 if individual j and i shared the same genotype on marker k; otherwise, φ_jik_was scored 0, and m was the number of markers. The haplotype-based CM (*C*_H_*M*) was applied for all markers as well, in which the number of haploblocks that were in the same state between pairs of individuals were counted. The genomic relatedness matrix in *C*_H_*M* is expressed as S_H_with entries $S_{\text{Hji}}=\left(\frac{\sum_{q-1}^{\text{Q}}\varphi_{\text{jiq}}}{Q_{\text{H}}}\right)$, where φ_jiq_ was scored 1 if individual i and j share the same haplotype allele configuration on haploblock q; otherwise, φ_jiq_ was scored 0; Q_H_ was the total number of haploblocks, which is the same with that in G_H_. Therefore, the entries of S_H_ represented the proportion of haploblocks with an identical state between pairs of individuals. For the five genomic classes, the haplotype-based CM assigned the genome annotation *C*_H_*M*|*GA* was applied. Similarly, the genomic relatedness matrix was built by counting the number of haploblocks that were in an identical state between pairs of individuals (Gao et al., 2017) and expressed as $\tilde{S}$ with entries ${\tilde{S}}_{\text{ji}}=\left(\frac{\sum_{q-1}^{\text{Q}}\varphi_{\text{jiq}}}{Q_{H_{\text{GA}}}}\right)$, whereφ_jiq_ is the same as in *C*_H_*M*, but $Q_{H_{\text{GA}}}$ is the total number of haploblocks in certain genomic class, which is the same with that in $G_{H_{\text{GA}}}$.

To capture the first-order epistasis among SNPs, the CM model can be extended to categorical epistasis (CE) model (Martini et al., 2017). In the CE model, the genotype combinations of each pair of loci were treated as categorical variables, and the relatedness of two individuals was measured by counting the number of pairs of markers in the same state. The genomic relatedness matrix in the CE model was be deduced from S via the formula*E* = 0.5 × *mS*#(*mS* + 1_n×n_)/*m*^2^, where # denotes the Hadamard product. The first-order epistasis between pairs of haploblocks was modeled by extending *C*_H_*M* to the haplotype-based categorical epistasis model (*C*_H_*E*) (Gao et al., 2017), where the genotype combinations of each pair of haploblocks were treated as a new categorical variable, and the genomic relatedness matrix was calculated as $E_{\text{H}}=0.5\times Q_{\text{H}}\,S_{\text{H}}\,\#\,\left(Q_{\text{H}}\,S_{\text{H}}+1_{n\times n}\right)/Q_{\text{H}}^{2}$. The corresponding epistatic model that included the first-order epistasis among haploblocks was developed for the five genomic classes and was denoted as *C*_H_*E*|*GA* (Gao et al., 2017), where the genomic relatedness matrix was constructed as $\tilde{E}=0.5\times Q\,\tilde{S}\,\#\,\left(Q\,\tilde{S}+1_{n\times n}\right)/Q_{H_{\text{GA}}}^{2}$.

### Assessment of Prediction Accuracy

The accuracy of GP was assessed using fivefold cross-validation (CV), which assigns animals randomly into five separate subsets with near-equal size. Each subset was used as the validation set only once, with phenotype masked, and the remaining four subsets were treated as a training set. In order to reduce random sampling effects, the CV layout described above was replicated twenty times, where a new randomization was implemented for each replicate so that the each of the subset contains different individuals. DGVs were calculated for each validation subset based on the genomic relatedness matrix. For each replicate, the prediction accuracies were assessed by the correlation between the DGVs and the pre-adjusted phenotypes in the validation set divided by square root of heritability. In addition, in order to assess the extent of bias on GP, linear regression coefficients [b (y, DGV)] of the pre-adjusted phenotypes (y) on the DGVs was calculated for individuals in the validation set. Unbiased models are expected to do not significantly different from 1, whereas values greater than 1 indicate a biased deflation prediction of DGVs and values smaller than 1 indicate a biased inflation prediction of DGVs.

## Results

### SNP Annotation and Heritability Estimation

We annotated 671,204 filtered SNPs into five genomic classes based on their physical positions. The annotation results and descriptive statistics of each genomic class are displayed in Table 4. Overall, 67.03 and 32.97% of the total SNPs were annotated into the IGR and gene classes, respectively. Only 1.46, 1.05, and 0.39% of the total SNPs were annotated into the exon, CDS, and UTR class, respectively. The average MAF among these five genomic classes was in the range of 0.25 to 0.26. The number of haploblocks of gene, exon, CDS, and UTR classes were 87,407, 45,748, 9287, 6799, and 2409, respectively. We counted the number of genome features that were annotated by SNPs for each genomic class (Table 4). For instance, 16,286 genes were annotated by SNPs in the gene class, representing 66.30% of the total genes in the bovine genome. Based on the GREML method, the heritability estimates of CW, LW, and SI, were 0.42, 0.38, and 0.40 respectively.

**TABLE 4:** Mapping results and statistical descriptions of each genomic classes.

Genomic class	# of SNPs^1^	MAF	Mean MAF (SD)	# of haploblocks	# of represented genome feature^2^
IGR class	449,918 (67.03%)	0.009–0.5	0.26 (0.15)	87,407	
Gene class	221,286 (32.97%)	0.009–0.5	0.26 (0.15)	45,748	16,286 (66.30%)
Exon class	9814 (1.46%)	0.010–0.5	0.25 (0.15)	9287	9287 (4.08%)
CDS class	7024 (1.05%)	0.010–0.5	0.25 (0.14)	6799	6799 (3.17%)
UTR class	2614 (0.39%)	0.010–0.5	0.25 (0.15)	2409	2409 (7.26%)
All markers	671,204	0.009–0.5	0.26 (0.15)	115,005	

*^1^The number of SNPs annotated in five genomic classes, and their percentage of the whole genome-wide markers is indicated in parentheses. ^2^The number of genomic features represented by SNPs in the corresponding genomic class, and their percentage of the total genome features of the reference genome in parentheses. The bovine reference genome contains 24,559 genes, 227,610 exons, 214,584 CDS, and 33,137 UTR. # means “the number.”*

### Prediction Accuracy of Haplotype-Based Prediction Model

**FIGURE 1:**
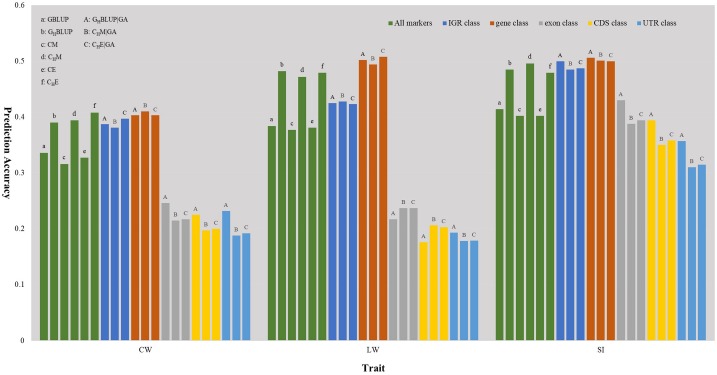
The prediction accuracies of different genomic classes in three traits of Chinese Simmental beef cattle.

We first compared the prediction accuracies of all markers between haplotype-based prediction models (*G*_H_*BLUP* and *C*_H_*M*) and the SNP-based prediction models (*GBLUP* and CM). The results showed that the predictive performances of *G*_H_*BLUP* and *C*_H_*M* were more accurate than *GBLUP* and CM in CW, LW, and SI (Figure 1). In the numerical dosage models, the accuracy of *G*_H_*BLUP* was 5.4, 9.8, and 7.1% higher than *GBLUP* in CW, LW, and SI, respectively (Table 5). In the CMs, *C*_H_*M* improved the accuracies by 7.8, 9.5, and 9.4% in CW, LW, and SI, respectively, compared with the CM results. Generally, the numerical dosage models performed better than CMs for most traits. For all markers, *GBLUP* slightly outperformed CM with 3.0, 0.7, and 1.2% higher accuracy in CW, LW, and SI, respectively (Table 5). The predictive performance of *G*_H_*BLUP* was 1% more accurate than *C*_H_*M* only in LW.

**TABLE 5:** The prediction accuracies (SD) of different genomic classes in three traits of Chinese Simmental beef cattle.

Trait^1^		Numerical dosage model		Categorical model		Categorical epistasis model	
CW	All maker	*GBLUP*	0.336 (0.05)	*CM*	0.316 (0.06)	CE	0.327 (0.06)
	All maker	G_H_*BLUP*	0.390 (0.06)	C_H_M	0.394 (0.06)	C_H_E	0.408 (0.06)
	IGR class	G_H_*BLUP*|*GA*	0.397 (0.06)	C_H_M|*GA*	0.387 (0.06	C_H_E|*GA*	0.381 (0.06)
	Gene class	G_H_*BLUP*|*GA*	0.403 (0.05)	C_H_M|*GA*	0.410 (0.06)	C_H_E|*GA*	0.403 (0.06)
	Exon class	G_H_*BLUP*|*GA*	0.246 (0.06)	C_H_M|*GA*	0.215 (0.05)	C_H_E|*GA*	0.217 (0.05)
	CDS class	G_H_*BLUP*|*GA*	0.225 (0.06)	C_H_M|*GA*	0.197 (0.05)	C_H_E|*GA*	0.200 (0.05)
	UTR class	G_H_*BLUP*|*GA*	0.232 (0.06)	C_H_M|*GA*	0.188 (0.05)	C_H_E|*GA*	0.192 (0.05)
LW	All maker	*GBLUP*	0.384 (0.05)	*CM*	0.377 (0.06)	CE	0.381 (0.06)
	All maker	G_H_*BLUP*	0.482 (0.06)	C_H_M	0.472 (0.06)	C_H_E	0.479 (0.05)
	IGR class	G_H_*BLUP*|*GA*	0.423 (0.06)	C_H_M|*GA*	0.425 (0.06)	C_H_E|*GA*	0.428 (0.06)
	Gene class	G_H_*BLUP*|*GA*	0.502 (0.07)	C_H_M|*GA*	0.494 (0.07)	C_H_E|*GA*	0.508 (0.07)
	Exon class	G_H_*BLUP*|*GA*	0.217 (0.06)	C_H_M|*GA*	0.237 (0.06)	C_H_E|*GA*	0.237 (0.06)
	CDS class	G_H_*BLUP*|*GA*	0.176 (0.06)	C_H_M|*GA*	0.206 (0.06)	C_H_E|*GA*	0.203 (0.06)
	UTR class	G_H_*BLUP*|*GA*	0.193 (0.06)	C_H_M|*GA*	0.178 (0.05)	C_H_E|*GA*	0.179 (0.05)
SI	All maker	*GBLUP*	0.414 (0.07)	*CM*	0.402 (0.07))	CE	0.402 (0.07)
	All maker	G_H_*BLUP*	0.485 (0.06)	C_H_M	0.496 (0.06)	C_H_E	0.479 (0.06)
	IGR class	G_H_*BLUP*|*GA*	0.487 (0.06)	C_H_M|*GA*	0.500 (0.06)	C_H_E|*GA*	0.485 (0.06)
	Gene class	G_H_*BLUP*|*GA*	0.506 (0.06)	C_H_M|*GA*	0.501 (0.06)	C_H_E|*GA*	0.500 (0.06)
	Exon class	G_H_*BLUP*|*GA*	0.430 (0.06)	C_H_M|*GA*	0.388 (0.06)	C_H_E|*GA*	0.394 (0.06)
	CDS class	G_H_*BLUP*|*GA*	0.394 (0.06)	C_H_M|*GA*	0.350 (0.05)	C_H_E|*GA*	0.358 (0.05)
	UTR class	G_H_*BLUP*|*GA*	0.357 (0.06)	C_H_M|*GA*	0.310 (0.06)	C_H_E|*GA*	0.315 (0.06)

*^1^Carcass weight (CW), live weight (LW), and striploin (SI); prediction accuracies are averaged over the fivefold cross-validation (CV) and then over the 20 replicates.*

### Prediction Accuracy of Haplotype-Based Prediction Model Given Genome Annotation

Under the haplotyped-based model, we further compared the prediction accuracies for the genomic classes with all markers to characterize the benefits of usage genome annotation information in GP. We found that the accuracy of using gene annotation to define haploblocks was consistently higher than that of all markers across all traits (Figure 1). In *G*_H_*BLUP*|*GA*, the prediction accuracy of gene class was 0.403, 0.502, and 0.506 for CW, LW, and SI, which were 1.3, 2.0, and 2.1% higher than using *G*_H_*BLUP*, respectively. In the CM, *C*_H_*M*|*GA* outperformed *C*_H_*M* in gene class, with accuracy improvements of 1.6, 2.2, and 0.5% in CW, LW, and SI, respectively. For IGR, exon, CDS, and UTR genomic classes, the accuracies using the two haplotype-based prediction models were not improved. In *G*_H_*BLUP*|*GA*, gene class had 0.6–7.9, 7.6–28.5, 11.2–32.6, and 14.9–30.9% higher accuracies than IGR, exon, CDS, and UTR classes for the three traits, respectively. Analogously, in *C*_H_*M*|*GA*, the accuracies of the three traits using gene class were 0.1–6.9, 11.3–25.7, 15.1–28.8, and 19.1–31.6% higher than that of IGR, exon, CDS, and UTR classes, respectively (Table 5). Comparing the prediction accuracy of numerical dosage with the CM, we found that *G*_H_*BLUP*|*GA* maintained more accurate predictive performance than *C*_H_*M*|*GA* in most genomic classes (Table 5).

### Prediction Accuracy of Epistasis Model

Considering the prediction model including epistatic effects may increase the accuracy and reduce the bias of DGVs. The results showed that incorporation of first-order epistatic effects into prediction model can slightly improve the prediction accuracies for most traits and genomic classes (Figure 1). When including the epistatic effects amongst SNPs into the CE model for all markers, prediction accuracy increased by 1.1 and 0.4% in CW and LW, respectively (Table 5). Similarly, the extension of *C*_H_*M* to *C*_H_*E* for all markers improved the prediction accuracies by 1.4 and 0.7% in CW and LW, respectively. For the five genomic classes, compared with *C*_H_*M*|*GA*, *C*_H_*E*|*GA* also had higher prediction accuracies in the IGR class of LW (0.3%), gene class of LW (1.4%), exon class of CW (0.2%) and SI (0.6%), CDS class of CW (0.3%) and SI (0.8%), and UTR class of CW (0.4%) and SI (0.5%).

### Regression Coefficient

Table 6 displayed the slope of the regression of the adjusted phenotype on DGVs. For numerical dosage models, the regression coefficients of all marker, IGR, and gene classes were not significantly different from 1 in all traits, indicating the predictions were not significantly biased. For CMs, the regression coefficients of gene, exon, CDS, and UTR classes were significantly different from 1 in CW and LW. However, the regression coefficients for the predictions using the CMs that included the first-order epistasis were significantly different from 1 in all markers and genomic classes, suggesting that these models increased the biasedness of GPs. Generally, among five genomic classes, the regression coefficients of IGR and gene classes were similar to those of all markers, and they contribute to less bias prediction than exon, CDS, and UTR classes. When compared haplotype-based prediction models without including epistasis to the corresponding SNP-based prediction models, we found that the formers’ regression coefficients were closer to one, with less biasedness prediction.

**TABLE 6:** Regression coefficients (SD) of pre-adjusted phenotypes on DGVs for three traits of Chinese Simmental beef cattle.

Trait^1^		Numerical dosage model		Categorical model		Categorical epistasis model	
CW	All maker	*GBLUP*	1.102 (0.08)	*CM*	**1.097** (0.05)	CE	1.087 (0.05)
	All maker	G_H_*BLUP*	1.062 (0.06)	C_H_M	1.079 (0.06)	C_H_E	**1.388** (0.08)
	IGR class	G_H_*BLUP*|*GA*	1.064 (0.06)	C_H_M|*GA*	**1.080** (0.07)	C_H_E|*GA*	**1.318** (0.07)
	Gene class	G_H_*BLUP*|*GA*	1.071 (0.06)	C_H_M|*GA*	**1.090** (0.06)	C_H_E|*GA*	**1.300** (0.07)
	Exon class	G_H_*BLUP*|*GA*	**1.131** (0.16)	C_H_M|*GA*	**1.143** (0.18)	C_H_E|*GA*	**1.135** (0.18)
	CDS class	G_H_*BLUP*|*GA*	**1.173** (0.18)	C_H_M|*GA*	**1.169** (0.23)	C_H_E|*GA*	**1.156** (0.21)
	UTR class	G_H_*BLUP*|*GA*	**1.165** (0.16)	C_H_M|*GA*	**1.232** (0.16)	C_H_E|*GA*	**1.218** (0.16)
LW	All maker	*GBLUP*	0.984 (0.10)	*CM*	1.062 (0.09)	CE	**1.094** (0.09)
	All maker	G_H_*BLUP*	1.009 (0.07)	C_H_M	1.023 (0.08)	C_H_E	**1.546** (0.10)
	IGR class	G_H_*BLUP*|*GA*	1.051 (0.07)	C_H_M|*GA*	1.073 (0.08)	C_H_E|*GA*	**1.311** (0.08)
	Gene class	G_H_*BLUP*|*GA*	1.051 (0.04)	C_H_M|*GA*	1.088 (0.04)	C_H_E|*GA*	**1.629** (0.04)
	Exon class	G_H_*BLUP*|*GA*	**1.187** (0.30)	C_H_M|*GA*	**1.159** (0.22)	C_H_E|*GA*	**1.165** (0.22)
	CDS class	G_H_*BLUP*|*GA*	**1.386** (0.31)	C_H_M|*GA*	**1.285** (0.25)	C_H_E|*GA*	**1.294** (0.25)
	UTR class	G_H_*BLUP*|*GA*	**1.197** (0.29)	C_H_M|*GA*	**1.282** (0.33)	C_H_E|*GA*C_H_E|*GA*	**1.278** (0.32)
SI	All maker	*GBLUP*	1.079 (0.03)	*CM*	1.076 (0.05)	CE	**1.083** (0.05)
	All maker	G_H_*BLUP*	1.038 (0.05)	C_H_M	1.046 (0.04)	C_H_E	**1.414** (0.07)
	IGR class	G_H_*BLUP*|*GA*	1.038 (0.05)	C_H_M|*GA*	1.049 (0.04)	C_H_E|*GA*	**1.338** (0.07)
	Gene class	G_H_*BLUP*|*GA*	1.050 (0.05)	C_H_M|*GA*	1.050 (0.05)	C_H_E|*GA*	**1.643** (0.06)
	Exon class	G_H_*BLUP*|*GA*	1.055 (0.03)	C_H_M|*GA*	1.048 (0.05)	C_H_E|*GA*	1.052 (0.05)
	CDS class	G_H_*BLUP*|*GA*	1.058 (0.05)	C_H_M|*GA*	1.046 (0.07)	C_H_E|*GA*	1.049 (0.08)
	UTR class	G_H_*BLUP*|*GA*	1.064 (0.07)	C_H_M|*GA*	1.081 (0.10)	C_H_E|*GA*	1.080 (0.10)

*^1^Carcass weight (CW), live weight (LW), and striploin (SI); for each trait (row), the values in bold face indicate the coefficient are significantly different from 1 (*p* < 0.05); regression coefficients are averaged over the fivefold cross-validation (CV) and then over the 20 replicates.*

## Discussion

Advances in high-throughput genotyping technology and the availability of genome annotation information have contributed to the improvement of the predictive performance of complex quantitative traits in livestock species (Morota et al., 2014; Do et al., 2015; Edwards et al., 2016; Nani et al., 2019). To bridge the gap between mathematical models and underlying biological processes, we combined bovine genome annotation information with haplotype-based prediction models to improve the predictive accuracies in Chinese Simmental beef cattle. In this study, whole genome-wide SNPs of BovineHD Beadchip were annotated to five genomic classes. The predictive performance of five genomic classes and all markers was assessed using both numerical and CMs, and the contribution of first-order epistatic effects among SNPs and haploblocks were modeled using categorical coding strategy.

### Predictive Performance of Haplotype-Based Prediction Model

Haplotypes have been used widely in human genetics research (Curtis et al., 2001; Chapman et al., 2003; Curtis, 2007); in animal breeding studies, haplotypes have been used for the GP of breeding values with the use of high density SNP chips (Calus et al., 2008; Boichard et al., 2012; Cuyabano et al., 2014; Mucha et al., 2019). In this study, haplotype-based prediction models (*G*_H_*BLUP* and *C*_H_*M*) were applied to the whole genome-wide markers, and the result of this scenario was treated as a benchmark. We found that the predictive performance of haplotype-based prediction models was superior to corresponding SNP-based prediction models in the three traits (Figure 1), with higher accuracy and less bias. This was consistent with previously reported results in simulated datasets (Calus et al., 2008; Villumsen et al., 2009), dairy cattle (Cuyabano et al., 2014; Hess et al., 2017; Karimi et al., 2018) and beef cattle (Hayes et al., 2007). This may be attributable to haplotypes better capturing LDs with causative mutation or QTLs than single SNPs.

In livestock, SNPs are commonly bi-allelic. When mutations occur, the allele frequencies may remain (almost) unaltered. However, mutations in different loci tend to cause major changes in the haplotype frequencies (Curtis et al., 2001). Thus, when haplotypes were analyzed, a QTL that was not in complete LD with any individual bi-allelic SNP marker may be in complete LD with a multi-marker haplotype. To use a haplotype as an indicator variable in GP, previous studies defined haploblocks by setting windows with a fixed number of SNPs to be placed together as a haploblock (Boichard et al., 2012; Schrooten et al., 2013; Hess et al., 2017), or by considering only the first locus out of 10 consecutive loci in genomic evaluation (Schrooten et al., 2013; Meuwissen et al., 2014). Although their prediction accuracies were improved in GP, the number of SNPs used to outline haploblocks was arbitrarily defined.

To efficiently use the genome properties to define haploblocks and reduce the number of variables for the GP models, several researchers used only haplotypes with a high frequency in the population (Mucha et al., 2019) or based on LD threshold to define haploblocks (Cuyabano et al., 2015). For instance, Cuyabano et al. (2014) used an average LD threshold (≥0.45) to construct haploblocks and found that prediction accuracies increased for the three traits compared with the commonly-used individual SNP. Similarly, we used the cattle genome annotation information to define a biologically functional unit and constructed a haploblock for each unit. This strategy may reflect underlying biological processes and avoid haploblocks being arbitrarily defined. Our study contributes to the improvement of prediction accuracy using a haplotype-based model, since the functional unit contains the combined effects of tightly linked *cis-*acting causal variants (Garnier et al., 2013; Da, 2015), and the number of haplotypes having effects was significantly larger than that for SNP models (Calus et al., 2008). Jiang et al. (2018) indicated that the increase in accuracy bringing by haplotype-based prediction models may be explained by this model capitalizing on local epistatic effects among markers.

### Predictive Performance Among Five Genomic Classes

In our study, we applied | GA approaches based on the concept of defining biologically functional units as predictor variables. The results showed that the accuracies and biasedness of prediction for gene and IGR classes were consistently better than those for the exon, CDS, and UTR classes, regardless of which | GA prediction models were used. Firstly, this finding may be attributed to the number of SNPs annotated in its corresponding genomic class, which decreased from the IGR to UTR classes. As previously suggested, the number of markers plays an important role in affecting the GP performance (Zhong et al., 2009; Daetwyler et al., 2010). With decreasing number of markers, the physical distance increased between the markers and QTLs and reduced the LD between markers and QTLs, which would lead to poor predictive power (Yang et al., 2010; Zhang et al., 2011; de los Campos et al., 2013). Yang et al. (2010) found that when the causative mutation loci had a lower MAF, a decrease in marker density would result in an incomplete linkage between the SNP and causative mutation loci; thus, these markers only explained a limited genetic variance.

In our study, 67.03 and 32.97% of the total SNPs were located within the IGR class and gene class, respectively, whereas only 0.39% of total SNPs was annotated in the UTR class, which had the lowest predictive accuracy. Secondly, the average number of SNPs in a haploblock may affect the prediction accuracy of genomic classes as well. It is clear that if each haploblock consisted of only one marker, the haplotype-based prediction models were exactly identical to the corresponding SNP-based prediction models (Gao et al., 2017). In the IGR and gene classes, 87,407 and 45,748 haploblocks were constructed (Table 4), respectively, and 96.82 and 94.21% of the total haploblocks consisted of more than one SNP, which resulted in 5.15 and 4.84 SNPs per haploblock on average, respectively. However, only 9287, 6799, and 2409 haploblocks were constructed in the exon, CDS, and UTR classes. The average number of SNPs per haploblock was 1.06, 1.03, and 1.08, respectively, which indicated haplotype-based prediction models for these genomic classes were similar to SNP-based prediction models. Finally, the number of biological functional units that was used to construct the statistical framework in the | GA approaches could also be a key factor in affecting the predictive accuracies, since the biological functional units may reflect the underlying biological process.

According to the bovine genome annotation information, the bovine reference genome contained 24,559 genes, 227,610 exons, 214,584 CDS, and 33,137 UTR. In this study, gene class represented 66.3% (16,286 out of 24,559 genes) of the total genes of the reference genome, whereas 4.08% (9287 out of 227,610 exons), 3.17% (6799 out of 214,584 CDS), and 7.26% (2409 out of 33,137 UTR) of the total exons, CDS, and UTR of reference genome were respectively represented by exon, CDS, and UTR classes. Consequently, the high proportion of biological-functional-unit-like genes may contribute to stronger predictive power. Taken together, these factors may explain the outstanding predictive performance displayed in gene class compared with the other classes.

### Benefits of Using Genome Annotation Information in GP

When the genome annotation information was incorporated into the haplotype-based prediction models, we also observed a slight or moderate improvement in prediction accuracies for the three traits. This can be explained by the traits having different genetic architectures (Daetwyler et al., 2010). The number of QTLs and the distribution of their effects may influence the prediction accuracies of genomic classes. For three traits, the gene class improved the prediction accuracy in comparison with the result of all markers using the haplotype-based prediction model, which was consistent with reported results in mouse and drosophila populations (Gao et al., 2017). This may reflect that genetic signals of the gene class are well tagged in these traits, despite more haploblocks being constructed in the scenario of all markers. The method of defining a biological unit through haplotypes might have increased the linkage of markers and QTLs, which not only allowed the effects of QTL to be better captured but also reduced the density of unrelated markers. Studies have reported that gene class has the most potential to be enriched for trait-associated variants and was more likely to explain a large proportion of the total additive variance (Kamanu et al., 2012; Kindt et al., 2013; Koufariotis et al., 2014). However, Morota et al. (2014) and Abdollahi-Arpanahi et al. (2016) found that the gene class did not lead to an improvement in predictive ability, and the whole genome-wide SNP-based prediction model remained the most efficient method for GP in chicken. These studies only annotated SNPs to the corresponding genomic class and applied the routine GP process for genomic classes. In this case, the genome annotation information cannot be comprehensively used in the SNP-based model because the biologically functional units were not defined as predictor variables in the model.

The usage of genome annotation information of the IGR class also led to a slight improvement in prediction accuracy in CW and SI. Studies have suggested that the IGR class, such as non-coding conserved regions, miRNA, and regulatory regions, might harbor important genetic variants associated with complex traits in crops (Hindorff et al., 2009; Schaub et al., 2012) and humans (Gusev et al., 2014; Finucane et al., 2015). For instance, a study suggested that more than 75% of identified SNPs are embedded in regulatory genome segments in common human diseases (Maurano et al., 2012). Therefore, the IGR class may contribute to a large phenotypic variation. Overall, combining the genome annotation information of the gene class with the haplotype-based prediction models can improve the prediction accuracies, and this can be considered as a promising tool of GP for economically important traits in Chinese Simmental beef cattle.

### Effects of Numerical and Categorical Model on Prediction Accuracy

When comparing the predictive performance of the numerical model with the CM, we found that *GBLUP* slightly outperformed the SNP-based CM in three traits. Martini et al. (2017) compared the predictive performance of *CM* with *GBLUP*, and found only slight differences in predictive ability between *CM* and *GBLUP* among 13 traits in mouse. The *CM* does not use the assumption of constant allele substitution effects like *GBLUP*; instead, it models the independent effect of each genotype at a locus, which enables the modeling of dominance (Martini et al., 2017). The advantages of *CM* depend on the population structure and the influence of the dominance effects on a particular trait. One reason to use *CM* instead of *GBLUP* might be the population having prevalent heterosis, since heterosis creates a deviation from the linear dosage model. When most loci are mainly present in only two of the three possible SNP genotypes, the CM cannot substantially outperform *GBLUP* (Martini et al., 2017). Gao et al. (2017) found that *G*_H_*BLUP* outperformed *C*_H_*M* in eight traits, and *C*_H_*M* outperformed *G*_H_*BLUP* in three traits. Analogously, in our study, *G*_H_*BLUP*|*GA* displayed better predictive performance than *C*_H_*M*|*GA* in most of the genomic classes among three traits. However, a similar pattern was not observed by Gao et al. (2017), who found that *C*_H_*M*|*GA* performed better than *G*_H_*BLUP*|*GA*in the gene class among most traits.

### Contribution of First-Order Epistasis to Prediction Accuracy

Epistasis has long been recognized as a biologically influential component contributing to the genetic architecture of quantitative traits (Mackay, 2014). Several genomic selection approaches have been developed to model both additive and epistatic effects (Xu, 2007; Cai et al., 2011; Wittenburg et al., 2011; Wang et al., 2012). To minimize the inherently high computational costs of those methods, EGBLUP (Jiang and Reif, 2015) and kernel Hilbert space regression accommodating epistasis within the GP models were proposed (Morota and Gianola, 2014). Generally, the influence of epistasis on GP ranges from positive to negative. In some studies, prediction accuracies increased (Hayes et al., 2009; Su et al., 2012; Jiang and Reif, 2015; He et al., 2016), whereas in others, modeling epistasis adversely affected prediction accuracies (Lorenzana and Bernardo, 2009). For instance, Su et al. (2012) extended GBLUP to EGBLUP to estimate both additive and additive by additive epistatic genetic effects. They found that the epistatic variance accounted for 9.5% of the total phenotypic variance, and the predictive reliabilities of genomic predicted breeding values increased by 0.3%, which was consistent with the results reported by Muñoz et al. (2014). These discrepancies can be explained by the complexities of the studied traits, which are controlled by many loci exhibiting small effects entailing a low QTL detection power.

In this study, the first-order epistatic effects were captured by the categorical epistasis model, which can eliminate the undesired coding-dependent properties of EGBLUP (He et al., 2015; Martini et al., 2017). Although EGBLUP has been applied in other studies (Jiang and Reif, 2015), Martini et al. (2017) suggested that both EGBLUP and the Gaussian kernel in an RKHS approach respond differently to a change in marker coding: a translation of the coding impacts the predictive ability of EGBLUP, but not that of the Gaussian kernel. The difference of coding strategy in the CM with the traditional encoding (0, 1, 2) in EGBLUP meant that the additivity assumption was not necessary in the categorical coding and the encoding of SNPs or haploblocks corresponded to the allele configurations, which enables the modeling of dominance (Martini et al., 2017). In CMs, for all markers, the first-order epistasis of pairs of SNPs were modeled by the CE model, and we found an increase in predictive accuracies from step CM to the CE model in all traits except SI. Martini et al. (2017) also found that CE was slightly better than CM in the simulated and mouse datasets.*C*_H_*E* modeling of the first-order epistasis between pairs of haploblocks also increased the predictive accuracies of all makers of CW and LW. Similarly, Gao et al. (2017) found an improvement in predictive ability from CM to CE, and from *C*_H_*M* to *C*_H_*E*. For genomic classes, we observed a slight increase in accuracy in the gene class of LW and the CDS class of SI from *C*_H_*M*|*GA* to *C*_H_*E*|*GA*. These findings suggest that the first-order epistatic effects captured by markers was likely to contribute to some of the phenotypic variations of the traits observed in this study.

## Conclusion

In our study, genome annotation information was incorporated into the haplotype-based prediction model for GP of three carcass traits in Chinese Simmental beef cattle. To enable comparison, the SNP-based and haplotype-based prediction methods were applied for all markers, and their results were treated as a benchmark. We found that when the haplotype was treated as a predictor variable, the prediction accuracy improved in most traits. After combining the genome annotation information of the gene class with the haplotype-based prediction model, a further increase in accuracy was observed in most traits compared with the results of all markers obtained by haplotype-based prediction models without genome annotation. The first-order epistatic effects among SNPs and haplotypes slightly improved the prediction accuracy of all markers in LW and CW. In conclusion, incorporating genome annotation information of gene classes into GP models through haplotype-based models could be considered as a promising tool for the GP of carcass traits in Chinese Simmental beef cattle.

## Data Availability Statement

Genotype data have been submitted to Dryad: doi: 10.5061/dryad.4qc06. Bovine genome annotation (Bos_taurus.ARS-UCD1.2) was downloaded from Ensemble (http://asia.ensembl.org/index.html).

## Ethics Statement

The animal study was reviewed and approved by Science Research Department of the Institute of Animal Sciences, Chinese Academy of Agricultural Sciences (CAAS) (Beijing, China).

## Author Contributions

LX simulated and analyzed the data and wrote the manuscript. ZW, LX, and YL collected the data. NG, YC, XG, HG, LYX, LZ, BZ, and JL discussed and improved the manuscript. All authors read and approved the final manuscript.

## Conflict of Interest

The authors declare that the research was conducted in the absence of any commercial or financial relationships that could be construed as a potential conflict of interest.

## Footnotes

1 http://asia.ensembl.org/index.html
